# Mouse cytomegalovirus lacking sgg1 shows reduced import into the salivary glands

**DOI:** 10.1099/jgv.0.002013

**Published:** 2024-08-02

**Authors:** Jiawei Ma, Kimberley Bruce, Philip G. Stevenson, Helen E. Farrell

**Affiliations:** 1 School of Chemistry and Molecular Biosciences, University of Queensland, Brisbane, Australia

**Keywords:** cytomegalovirus, myeloid cells, salivary glands

## Abstract

Cytomegaloviruses (CMVs) transmit via chronic shedding from the salivary glands. How this relates to the broad cell tropism they exhibit *in vitro* is unclear. Human CMV (HCMV) infection presents only after salivary gland infection is established. Murine CMV (MCMV) is therefore useful to analyse early infection events. It reaches the salivary glands via infected myeloid cells. Three adjacent spliced genes designated as m131/129 (MCK-2), sgg1 and sgg1.1, positional homologues of the HCMV UL128/130/131 tropism determinants, are implicated. We show that a sgg1 null mutant is defective in infected myeloid cell entry into the salivary glands, a phenotype distinct from MCMV lacking MCK-2. These data point to a complex, multi-step process of salivary gland colonization.

## Full text

Human cytomegalovirus (HCMV) is a ubiquitous human herpesvirus which transmits chiefly via saliva [1]. HCMV seroconversion usually occurs in childhood [2] and is asymptomatic in immunocompetent hosts, making it difficult to track. Symptomatic infection in vulnerable hosts is the result of systemic spread, with saliva testing providing a sensitive readout of active infection [3]. As cytomegaloviruses (CMVs) long predate human speciation, features of host colonization and spread are unlikely to be unique [4]. Thus, natural animal models can provide insight. Infection of mice with murine CMV (MCMV) is a tractable animal model to trace virus spread *in vivo*. MCMV enters new hosts via olfactory neurons [5]; infected myeloid cells then carry it systemically, and salivary gland (SG) acinar epithelial cells support chronic shedding [6,7].

The species specificity of the CMVs limits *in vivo* studies of HCMV tropism. *In vitro* data support two modes of cell-free HCMV entry: a pH-independent plasma membrane fusion via a trimer comprised of glycoproteins H, L and O (gH/gL/gO) and a pH-dependent endocytic entry facilitated by a pentameric complex (PC) comprised of gH/gL with products of the UL128 locus (UL128, UL130 and UL131A) [8,9]. Mutation studies show that the trimeric gH/gL/gO trimer and PC likely co-operate in a multi-step pathway [10]. An MCMV gH/gL/MCK-2 trimer complex has been identified and linked to tropism for myeloid cells [11,13]. MCK-2 is a positional homologue of HCMV UL128, and like UL128, it shows homology with cellular C–C chemokines, although no sequence conservation exists between them [14,15]. *In vivo*, MCK-2 was dispensable for extravasation of CD11c^+^ myeloid cells to the SG but was critical for subsequent infection of acinar epithelial cells [12].

Spliced genes sgg1 and sgg1.1 are also positional homologues of the UL128 locus. A role of sgg1 in the SG tropism was inferred from mutants bearing either large deletions or insertions which may have interfered with the expression of neighbouring transcripts [16,18]. Later analysis identified sgg1.1 as a spliced gene overlapping the main deletion mutant used [19]. A point mutation in sgg1.1 also reduced SG infection, raising the possibility that its disruption contributed to the phenotype identified for sgg1. To resolve this question and understand how sgg1 might work, we introduced a premature translation stop into its exon 1, thereby preserving sgg1.1.

**Fig. 1.**
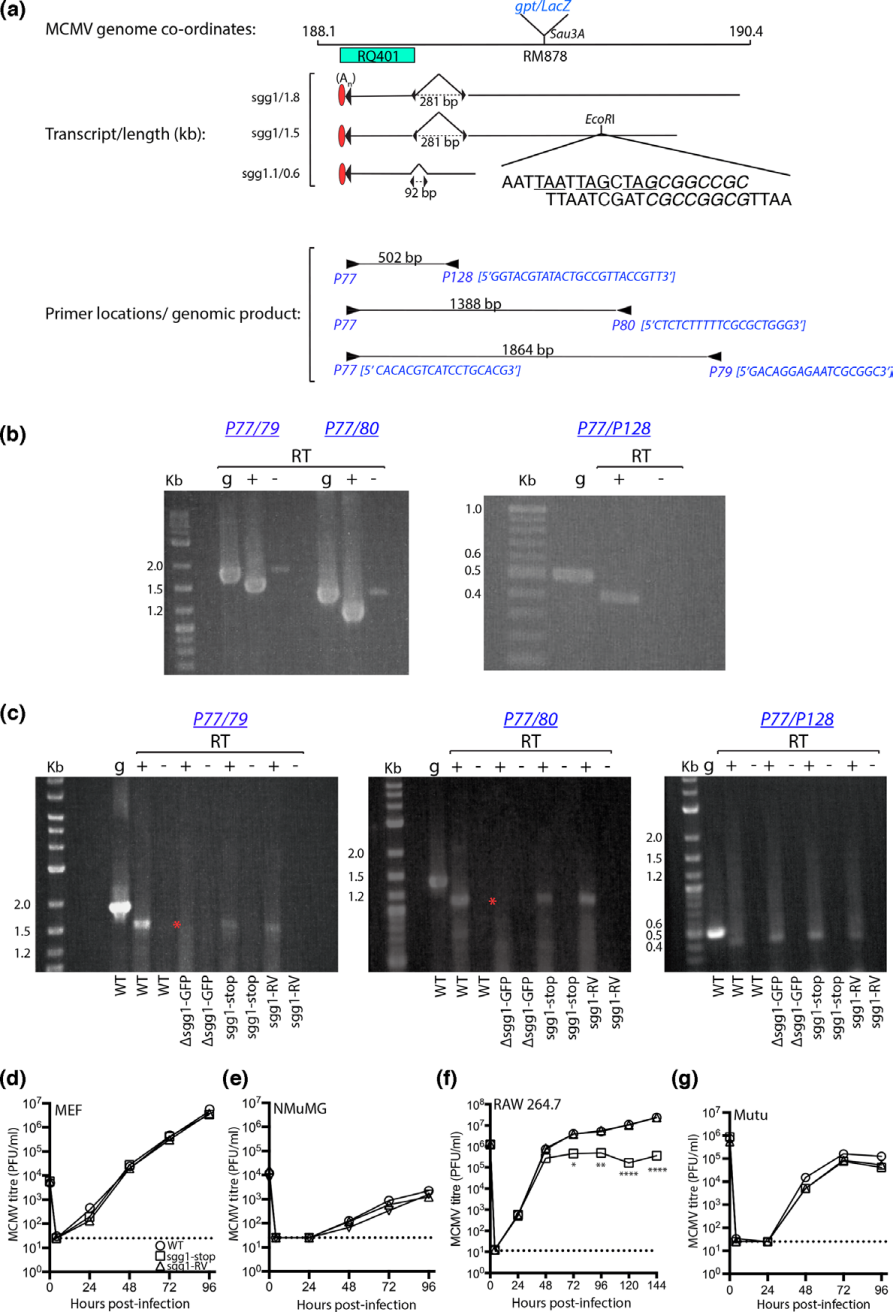
Organization of the sgg1 locus. The location and orientation of spliced transcripts in the sgg1 region of the MCMV genome are shown in (**a**). Three spliced transcripts have been identified to date, including sgg1, which comprises two transcripts (1.8 kb and 1.5 kb), the sgg1.1 0.6 kb transcript. All three transcripts share a common 3′ untranslated region and polyadenylation [A(n)] signal. The sequence of the three-frame premature stop codons inserted in the *EcoR*I site of sgg1 (genomic location 189.7 kb) is shown, with the stop codons underlined. The inserted sequence included a unique *NotI* site (italics) for diagnostic purposes. The 5′−3′ sequence of primers P77, P79, P80 and P128 and their locations are shown, together with their predicted size when amplified from genomic DNA. The location of the 323 bp deletion of mutant RQ401 [16] and the Sau3A site for insertion of a gpt/LacZ expression cassette [17] are depicted. The *EcoR*I site used in this study for the insertion of a GFP expression cassette or three-frame translational stop codon is also shown. Amplified products of WT sgg1 and sgg1.1 transcripts, performed in the presence (+) or absence (−) of RT is shown in (**b**). PCR products from genomic (g)DNA are also shown to indicate unspliced products. Low abundance products amplified by primers P77/79 and P77/80 are consistent with gDNA contamination in the RNA preparations. Products amplified from the sgg1 locus from cultures infected with either WT, Δsgg1-GFP, sgg1-stop or sgg-RV are shown in (**c**). RT-PCR reactions were conducted in the presence (+) or absence (−) of RT, and gDNA was used as a control for unspliced product. The disruption of 1.8 and 1.5 kb sgg1 transcripts in cultures infected with Δssg1-GFP is indicated by an asterisk. Size markers in kilobases are shown in all images. Multi-step growth of WT, sgg1-stop and sgg1-RV in MEF (**d**), NMuMG (**e**), RAW 264.7 cells (**f**) and Mutu cells (**g**). MEF and NMuMG cultures were infected at an MOI of 0.05 for 1 h at 37 °C. Following the incubation, the inoculum was removed, the cultures washed three times with warm medium and then incubated up to 4 dpi. Cultures were frozen (−80 °C) at the indicated hours p.i. (*n*=3 per time point) and then plaque-assayed for infectious virus. RAW 264.7 and Mutu cultures were infected similarly, except at an MOI of 5.0 (relative to MEF), were washed with low pH medium (pH=5.0) to remove unbound virus and were sampled up to 144 h p.i. Titres are expressed in plaque-forming units per ml (PFU/ml); the dotted line indicates the limit of detection by the plaque assay. Comparisons were performed using a two-way ANOVA with Bonferroni’s multiple comparison test; *P<0.05; **P<0.01; ****P<0.0001.

Translational three-frame stop codons were introduced into sgg1 of MCMV strain K181 carrying a natural mutation in the m157 NK interaction gene (kindly provided by Alec Redwood, University of Western Australia) [20]. The genomic location of sgg1/sgg1.1, the sgg1 1.5/1.8 kb and sgg1.1 0.6 kb spliced transcripts, the location of the published deletion/insertion and the premature stop codons introduced into the *EcoR*I site of sgg1 is shown in Fig. 1a [16,17]. We first confirmed the reported 1.8 kb/1.5 kb sgg1 transcripts using RNA purified from mouse embryonic fibroblasts (MEF) infected 24 h previously with WT MCMV. cDNA was prepared using oligo-d(T) in the presence (+) or the absence (−) of reverse transcriptase (RT) and products amplified using primers shown in Fig. 1a. RT polymerase chain reaction (RT-PCR) products of the predicted size were generated for all three transcripts (Fig. 1b) and were reduced in size compared with PCR products from genomic DNA (designated ‘g’), consistent with splicing [16,17, 19]. Products of low abundance detected in RT-negative controls are a size consistent with genomic DNA detection.

To make MCMV with sgg1 disrupted selectively, a HCMV IE1 promoter-driven GFP expression cassette was inserted at an *EcoR*1 site within sgg1 exon 1 to generate Δsgg1-GFP (Fig. 1a) [15]. This was then repaired with WT sgg1 sequences containing a three-frame translation stop in sgg1 (designated sgg1-stop). As a control, we also repaired Δsgg1-GFP with WT sgg1 sequence to generate a sgg1 revertant (designated sgg1-RV). The sgg1-stop and sgg1-RV mutants were prepared by homologous recombination [21] using Δsgg1-GFP as the parent virus; GFP^−^ recombinants were plaque purified and the sgg1 locus sequenced to confirm their identity (data not shown).

We compared the sgg1 and sgg1.1 spliced transcripts produced in MEF infected with either WT MCMV, Δsgg1-GFP, sgg1-stop or sgg1-RV using the above procedures. We detected the correctly sized RT-PCR products for WT MCMV, sgg1-stop and sgg1-RV (Fig. 1c). As expected, both sgg1 transcripts were absent for Δsgg1-GFP (highlighted with an asterisk in Fig. 1c). Importantly, sgg1 and sgg1.1 transcripts in sgg1-stop and sgg1-RV mutants were preserved. We focused on the replication of GFP^−^ recombinant viruses.

We compared the growth of WT, sgg1-stop or sgg1-RV in MEF, epithelial NMuMG cells (ATCC CRL-1636) and macrophage RAW 264.7 (ATCC TIB-71) and mouse dendritic Mutu [22] cell lines, which were cultured as described previously [12]. Virus growth in MEF, NMuMG and Mutu cells were equivalent between viruses (Fig. 1d and e). In RAW 264.7 cells, virus titres were equivalent at 48 h p.i. (Fig. 1f), but sgg1-stop then selectively failed to amplify further, indicating a reduced capacity to propagate between RAW 264.7 cells. It should be noted that RAW 264.7 and Mutu cell infection is in general less productive for MCMV than MEF or NMuMG infection.

**Fig. 2.**
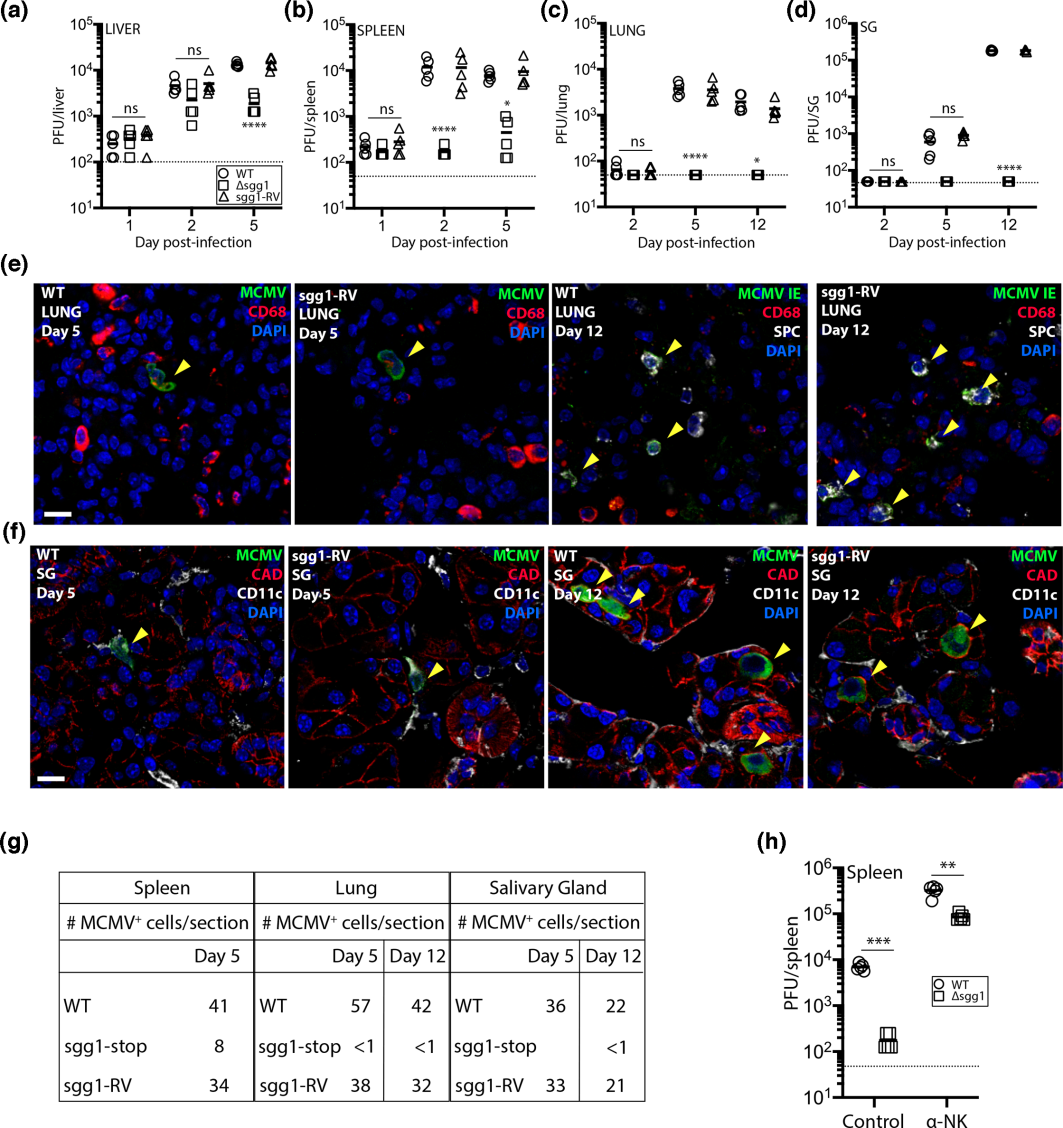
Sgg1 facilitates acute and persistent infection following i.p. inoculation. Six-week-old female BALB/c mice were inoculated i.p. with 10^6^ PFU WT, sgg1-stop or sgg1-RV (0.1 ml; *n*=5 mice/group). The infectious titres of the livers (**a**), spleens (**b**), lungs (**c**) and SGs (**d**) were determined by plaque assay on the dpi indicated. Example staining of lungs from WT and sgg1-RV-infected mice taken 5 and 12 dpi is shown in (**e**). Lungs were stained for MCMV infection (either rabbit polyclonal antibody to MCMV prepared in-house for lungs taken at 5 dpi or mouse MAb CROMA101 to MCMV immediate-early (IE) antigen at 12 dpi); CD68^+^ monocytes/macrophages (rat Mab FA-11; Abcam) and surfactant protein C^+^ (SPC^+^) type II alveolar epithelial cells (AECII) (rabbit Mab EPR19839; Abcam) on 6 µm frozen lung sections, followed by Alexa Fluor conjugated goat anti-rabbit/goat anti-mouse IgG_1_^488^, goat anti-rat IgG^568^ or goat anti-rabbit IgG^647^. Sections were counterstained with 4′,6′-diamidino-2-phenylindole (DAPI). Arrows show MCMV^+^CD68^+^ cells at 5 dpi and MCMV IE^+^SPC^+^ cells at 12 dpi. No MCMV^+^ cells were detected in the lungs of mice infected with sgg1-stop (not shown). Immunostaining for MCMV-infected cells (stained with polyclonal rabbit antiserum), CD11c^+^ cells (hamster Mab HL-3; BD Pharmingen) and E-cadherin^+^ cells (CAD; goat polyclonal antibody R and D Systems) in the SGs of mice infected with WT or sgg1-RV taken 5 and 12 dpi is shown in (**f**). Secondary antibodies included Alexa Fluor–conjugated anti-rabbit^488^/anti-goat^568^ and anti-hamster^647^ (all from Abcam). Sections were counterstained with DAPI. Arrows show MCMV^+^CD11c^+^ cells at 5 dpi and MCMV^+^CAD^+^ cells at 12 dpi. The scale bar is 20 µM. No MCMV^+^ cells were detected in the lungs of mice infected with sgg1-stop. The number of MCMV^+^ cells in frozen sections of spleens, lungs and SGs (*n*=5 tissues per section; minimum of 20 sections counted) taken on the dpi with either WT, sgg1-stop or sgg1-RV is shown in (**g**). BALB/c 6-week-old female mice were treated or not with anti-asialo GM_1_ (Wako Pure Chemicals; dose administered according to manufacturer’s instructions) on days −3, –1 and +1 relative to i.p. MCMV infection on day 0 with either sgg1-RV or sgg1-stop (10^6^ PFU). Virus titres in the spleens (*n*=5 mice/group) determined by plaque assay at 3 dpi are shown in (**h**). Comparisons between antibody-treated and control mice were performed by Student’s t-test; **P<0.01; ***P<0.001.

We then analysed sgg1 infection *in vivo*, to determine if sgg1-stop had an *in vivo* phenotype independent of that reported for sgg1.1. We infected BALB/c mice with sgg1-stop, sgg1-RV or WT MCMV intraperitoneally (i.p.) and tracked spread to the spleen, liver, lungs and SGs. Colonization of the liver and spleen 1 day post-infection (dpi) was equivalent between all viruses (Fig. 2a and b), but while WT and sgg1-RV infections then amplified, sgg1-stop infection was significantly less. It should be noted that sgg1.1 is not required for normal spleen and liver infection after i.p. inoculation of BALB/c mice [19]. We detected WT and sgg1-RV spread to the lungs and SGs at 5 and 12 dpi but failed to detect infection in these sites by sgg1-stop (Fig. 2c and d).

Histological analysis of the lungs and SGs confirmed a severe defect of sgg1-stop, with few infected cells. At 5 dpi, most MCMV^+^ cells in WT- and sgg1-RV-infected lungs were CD68^+^ myeloid cells. By 12 dpi, most were surfactant protein C^+^ (SPC^+^) epithelial cells (Fig. 2e). At 5 dpi in the SGs, most WT- and sgg1-RV-infected cells were CD11c^+^; by 12 dpi, most were e-cadherin^+^ acinar epithelial cells, consistent with the previous studies [12].

Quantification of MCMV^+^ cells in the spleen, lung and SGs also confirmed plaque assay data with significantly fewer positive cells detected for sgg1-stop than for WT or sgg1-RV (Fig. 2g). MCMV does not spread systemically from the liver [23], and while reduced spleen infection by sgg1-stop might have made some contribution to its reduced lung and SG infection, we have found previously that an i.p.-inoculated MCK-2 null mutant to have no defect in lung infection despite an equivalent defect in spleen infection [12,15].

Reduced early splenic infection by i.p.-inoculated MCMV has previously been linked to impaired NK cell evasion [24]. The setting for this was C57BL/6 mice infected with m157^+^ MCMV [25,26]. Nonetheless, NK cells also contribute – albeit rather less − to MCMV control in BALB/c mice and without m157 [27]. Therefore, to test whether NK cells might contribute to the attenuation ofsgg1-stop, we inoculated sgg1-stop or sgg1-RV i.p. into BALB/c mice treated or not with anti-asialo GM1. Antibody-treated mice had higher spleen titres of both sgg1-stop and sgg1-RV at 3 dpi, consistent with control by NK cells. However, spleen titres remained significantly lower in sgg1-stop-infected mice than in sgg1-RV-infected controls (Fig. 2h). Therefore, acute sgg1-stop attenuation in the spleen was not due to enhanced NK cell attack.

The spleen is not a significant tropism target following natural, olfactory MCMV infection [5]. Nevertheless, inoculations such as the i.p. route provide additional tissue contexts to probe viral gene function; the detection of a chemokine-like role for MCK-2 which is independent of its tropism function is an example (reviewed by Eletreby *et al.* [11]). Based on i.p. infection, a possible role for the rapid and early attenuation in the spleen is that sgg1 acts as a potent inhibitor of apoptosis. Splenic fibroblasts and dendritic cells within the marginal zone are the major early targets for lytic MCMV following i.p. infection [28], and while we did not detect an attenuated sgg1 phenotype for fibroblast infection *in vitro*, pro-apoptotic mediators released in the splenic milieu may uncover an anti-apoptotic function for sgg1, similar to that described for MCMV M36 [29].

**Fig. 3.**
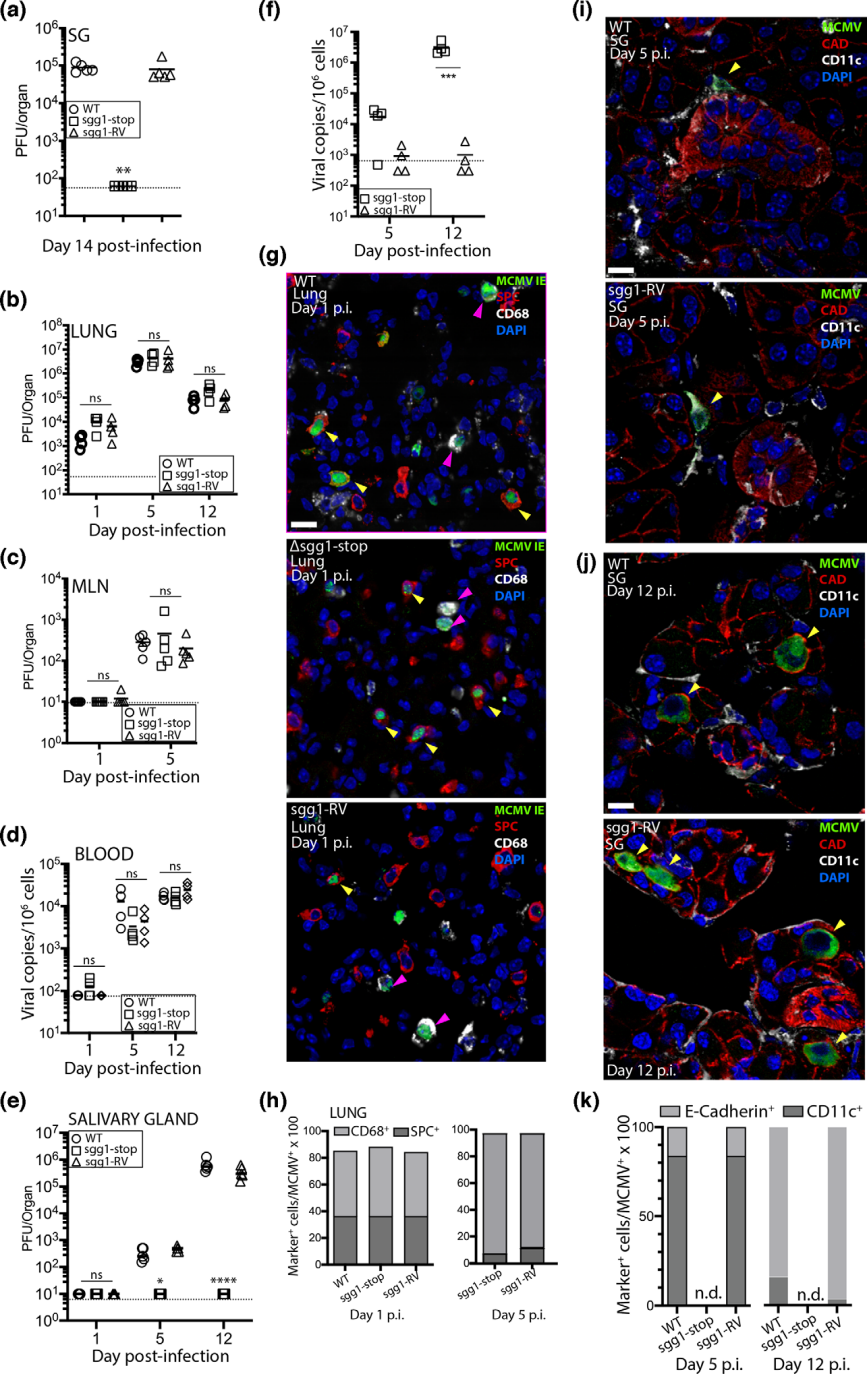
MCMV ssg1 gene promotes import of infected myeloid cells to the SGs. The nares of alert BALB/c female 6-week-old mice were administered with 5×10^4^ PFU of WT, sgg1-stop or sgg1-RV (4 µl inoculum; *n*=4 mice/group). SGs were harvested 14 dpi, and infectious titres from homogenates plaque assayed are shown in (**a**). Symbols indicate titres of individual mice; bars show means. The dashed line indicates the limit of detection. Comparisons were performed using ANOVA with Tukey’s multiple comparison. **P<0.01. Isofluorane-anaesthetized BALB/c mice were infected intranasally with 10^6^ PFU WT, sgg1-stop or sgg1-RV (30 µl; *n*=4/group). Virus titres in the lungs (**b**), mediastinal lymph node (MLN) (**c**), blood (**d**) and SG (**e, f**) were determined by either plaque assay (giving readout of PFU/organ) or quantitative PCR (qPCR; giving readout of copies/10^6^ cells) on the days indicated. The qPCR assay has been previously described [7]. Symbols indicate values from individual mice; bars show means. The dashed line indicates the limit of detection. Comparisons were performed using ANOVA with Tukey’s multiple comparison. *P<0.05; ****P<0.0001. Immunohistochemical staining of the lungs 1 dpi of BALB/c mice with either WT, sgg1-stop and sgg1-RV is shown in (**g**). Staining was performed using primary antibodies as for Fig. 2 to detect MCMV IE antigen^+^, CD68^+^ and SPC^+^ cells in the lungs. Secondary antibodies included Alexa Fluor–conjugated goat anti-mouse IgG_1_^488^, goat anti-rabbit IgG^568^ and goat anti-rat IgG^647^. Sections were counterstained with DAPI. Yellow arrows show MCMV IE^+^/SPC^+^ cells; pink arrows show MCMV IE^+^/CD68^+^ cells. The relative proportion of MCMV^+^ CD68^+^ and AECII^+^ cells in the lungs 1 and 5 dpi is shown in (**h**); at least 100 MCMV^+^ cells were counted. SGs harvested at 5 dpi (**i**) and 12 dpi (**j**) were stained for MCMV^+^/CAD^+^/CD11c^+^ cells using the same approach as described in Fig. 2. Yellow arrows show MCMV^+^/CD11c^+^ cells at 5 dpi and MCMV+/CAD +cells at 12 dpi. At least 100 MCMV^+^ cells, from up to 20 sections, were evaluated. The scale bar is 20 µM. The relative proportion of MCMV^+^ CAD^+^ and CD11c^+^ cells in the SGs 5 and 12 dpi is shown in (**k**). At least 100 MCMV^+^ cells, from up to 20 sections, were counted.

To test sgg1 function via olfactory acquisition, we infected the nares of BALB/c mice with either WT MCMV, sgg1-stop or sgg1-RV and quantified virus spread to the SGs 14 dpi. MCMV was detected in all SGs of mice infected with either WT or sgg1-RV, but not in mice infected with sgg1-stop (Fig. 3a). As nasal MCMV inoculation reaches the SGs without marked infection of the liver or spleen [5], this provided further evidence that the SG phenotype of sgg1-stop was independent of its splenic phenotype.

Nasal infection showed that the sgg1 defect was physiologically relevant. However, mice retain very little fluid volume here, so experimental virus delivery here is somewhat variable and systemic spread asynchronous between individuals. MCMV spreads similarly from the lungs – via infected CD11c^+^ myeloid cells – and they are easier to inoculate consistently, so we used lung infection to identify where in host colonization sgg1 acted.

We infected mice with WT, sgg1-stop or sgg1-RV MCMV and quantified virus loads in lung homogenates by plaque assay at 1, 5 and 12 dpi. We quantified viraemia by qPCR of blood at 1 and 5 dpi. In contrast to lung infection via the blood after i.p. MCMV, inhaled sgg1-stop MCMV reached lung titres equivalent to WT (Fig. 3b). Spread to the MLN and blood was also equivalent (Fig. 3c, d). However, sgg1-stop was not detectable in the SGs at either 5 or 12 dpi.

In the lungs, MCMV infects type II alveolar epithelial cells (AECII) as well as CD68^+^/CD11c^+^ myeloid cells [7,30]. To look for an effect of sgg1 on tropism, we stained lungs 1 dpi for CD68 and CD11c to mark myeloid cells, SPC to mark AECII and the MCMV immediate-early nuclear protein to mark active infection [using monoclonal antibody (MAb) CROMA 101, kindly provided by Stipan Jonjic, University of Rijeka] (Fig. 3g). Counting IE1^+^ cells revealed no difference between the viruses at days 1 and 5 p.i., with most infected cells being myeloid (CD68^+^ and/or CD11c^+^) (Fig. 3h).

Immunohistochemical staining of infected SGs showed WT and sgg1-RV MCMV to be >80 % in CD11c^+^ cells at 5 dpi and >90 % in e-cadherin^+^ cells at 12 dpi (Fig. 3j, k), consistent with blood-borne CD11c^+^ myeloid cells transferring infection to acinar epithelial cells [12]. We did not find MCMV^+^ cells in any of the >20 sections (three SGs/section) from sgg1-stop-infected mice on both dpi (not shown). Thus, sgg1 appeared to be necessary for infected CD11c^+^ cells to enter the SGs.

MCK-2, sgg1 and sgg1.1 genes are positional homologues of HCMV UL128/UL130/UL131A and similarly arranged [31,32] suggesting functional analogy [33], much as MCMV m74 is a positional homologue of HCMV UL27 (gO) and is functionally analogous *in vitro*, despite considerable sequence divergence [34,35]. Whether sgg1 and sgg1.1 associated with gH is however not yet testable, due to a lack of relevant antibodies.

In general, MCMV matches HCMV in having a broad tropism and disseminating via myeloid cells [36,37]. Tracking MCMV spread using a combination of plaque assay, immunohistochemistry and qPCR readouts has helped to identify viral proteins important for acute and persistent infection. MCMV proteins in addition to MCK-2, sgg1 and sgg1.1 likely contribute to *in vivo* spread. The MCMV G protein-coupled receptor designated M33, analogous to HCMV UL33 and possessing a constitutive signalling profile similar to the HCMV C–C chemokine receptor, US28 [7,21, 38, 39], uses differential G_q_/G_i/o_ coupling to facilitate traverse of MCMV^+^ CD11c^+^ cells from lymph nodes to the blood and, subsequently, for their extravasation to the SGs [7,40, 41]. Sgg1 evidently acts in the latter context; its role in the SG import also seems different to the role of MCK-2. Potentially, multiple viral glycoproteins might help disseminate cells to adhere to vascular endothelium for extravasation. Of note, we have seen no evidence of vascular endothelial infection by MCMV *in vivo*; a report of MCMV infecting vascular endothelium was based entirely on tunica intima endothelial kinase 2 (TIE2)-driven expression of a cre transgene, which is now known to be expressed also in haemopoietic cells [42,43]

One possible explanation for the involvement of multiple viral proteins is that spread is likely more complicated than direct passage of myeloid cells from draining lymph nodes to the SGs. Notably, acute viral replication in olfactory tissues is very limited, so amplification in peripheral tissues seems needed to establish a substantial SG infection [44]. Viral luciferase tracking showed neonatal infection seeding acutely to many sites before settling in the SGs [5], and tracking i.p.-inoculated MCMV showed that infected myeloid cells can potentially reach many sites [45]. A need to amplify in other tissues would also fit the fibroblast/epithelial tropism switch described for HCMV. However, in the absence of matching *in vitro* tropism effects for MCMV, it remains speculative. Severe splenic attenuation of sgg1^−^ MCMV may indicate an additional role for sgg1, although how it might impact virus spread via natural olfactory infection is unknown. The obvious next steps are further analysis of MCMV gH partners, localizing the host colonization defect of sgg1.1 mutants and developing reporter-based assays to track acute MCMV dissemination to non-salivary tissues. The latter is of particular interest as such dissemination seems the base of HCMV-driven disease.

## Abbreviations

AECII: type II alveolar epithelial cells
CMV: cytomegalovirus
DAPI: 4',6′-diamidino-2-phenylindole
dpi: day post-infection
gH: glycoproteins H
gL: glycoproteins L
gO: glycoproteins O
HCMV: human CMV
IE: immediate-early
i.p.: intraperitoneally
MAb: monoclonal antibody
MCMV: murine CMV
MEF: mouse embryonic fibroblasts
MLN: mediastinal lymph node
PC: pentameric complex
PFU: plaque forming units
qPCR: quantitative PCR
RT: reverse transcriptase
RT-PCR: RT polymerase chain reaction
SG: salivary gland
SPC^+^: surfactant protein C^+^
